# Supplementary material for: Evidence for Range Expansion and Origins of an Invasive Hornet Vespa bicolor (Hymenoptera, Vespidae) in Taiwan, with Notes on Its Natural Status
Source: Insects. 2021 Apr 2;12(4):320. doi: 10.3390/insects12040320 (PMC8066726; doi:10.3390/insects12040320)
Supplement: Supplementary file 1 [file insects-12-00320-s001.pdf]

```
          6911145661111111112222222222222333333333344444445555555555566666
            23897060336678890113344456889111233457900258990234444589900013
                   8282510981034803687251258239815328630287320369554603963  N
Hap_1     ATTCACATCCTTCAATTCCTTCTGCTCTTAAGTCTTAACTCCCCTCCTTTCATTTTTACAGCTT  2
Hap_2     ..A...GCTT..T..C.T.C.T...C.C.GTA.T.CT.....TT.TA.........C.....C.  1
Hap_3     GA.T.TG.T.C.TTGC.TT...CA.C..CG.A.T....T..TATC.TCC.TG.CC..TT.AT.C 23
Hap_4     G...GTC.TTCAT...CATCCTCC.CT....ACT...CTAT.T..TT..CTG.C.CC..GT.C.  1
Hap_5     GA.T.TG.T.C.TTGC.TT...CATC..CG.A.TC...T..TATC.TCC.TGCCC..TT.AT.C  1
Hap_6     GA.T.TG.T.C.TTGC.TT...CA.C..CG.A.T....T..TATC.TCC.TGCCC.CTT.A..C  1
Hap_7     ...............................AC..........................GA...  1
```

Figure S1. Sequence variation of 7 haplotypes of V. *bicolor* in the COI region. Haplotype sample from: Hap_1: Vietnam, Hap_2: Beijing, Hap_3: Taiwan, Hap_4: Nepal, Hap_5: Huangshan, Hap_6: Hong Kong, Hap_7: Vietnam. Dots "." denote identity with the reference sequence. The number of individuals sharing the same haplotypes is indicated in the right column by "N".

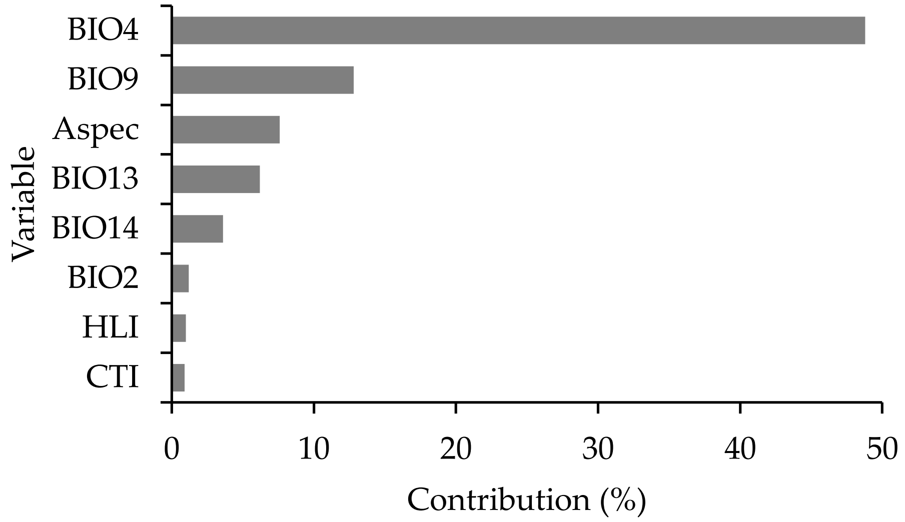

Figure S2. Variables contribution for modeling.

Table S1. List of environmental variables used in the model development.

Variables	Code
Annual mean temperature	BIO1
Mean diurnal range (Mean of monthly (max temp - min temp))	BIO2
Isothermality (BIO2/BIO7) (×100)	BIO3
Temperature seasonality (Standard deviation×100)	BIO4
Maximum temperature of warmest month	BIO5
Minimum temperature of coldest month	BIO6
Temperature annual range (BIO5 - BIO6)	BIO7
Mean temperature of wettest quarter	BIO8
Mean temperature of driest quarter	BIO9
Mean temperature of warmest quarter	BIO10
Mean temperature of coldest quarter	BIO11
Annual precipitation	BIO12

Precipitation of wettest month	BIO13
Precipitation of driest month	BIO14
Precipitation seasonality (Coefficient of variation)	BIO15
Precipitation of wettest quarter	BIO16
Precipitation of driest quarter	BIO17
Precipitation of warmest quarter	BIO18
Precipitation of coldest quarter	BIO19
Elevation	Eleva
Slope	Slope
Aspect	Aspec
Compound topographic index	CTI
Heat load index	HLI
